# Advantages and Disadvantages of the Arthroscopic Procedure in Acromioclavicular Separation

**DOI:** 10.3390/jcm14207130

**Published:** 2025-10-10

**Authors:** Mihai Tudor Gavrilă, Vlad Cristea, Stefan Cristea

**Affiliations:** 1 Department of Orthopedics and Traumatology, St Pantelimon Emergency Hospital, 021659 Bucharest, Romania; drstefancristea@yahoo.com; 2 Department of Orthopedics and Traumatology, Colentina Hospital, 020125 Bucharest, Romania; vladcristea@hotmail.com

**Keywords:** acromioclavicular separation, shoulder, clavicle, scapula, surgery, arthroscopy

## Abstract

Arthroscopic treatment of acromioclavicular (AC) joint separations has evolved significantly over the past two decades. Modern anatomical repair methods frequently rely on suspensory fixation devices to reconstruct the coracoclavicular ligaments and, in some cases, to stabilize the AC joint itself. **Background/Objectives**: Arthroscopy offers a minimally invasive option that ensures excellent visualization of the joint, facilitates management of concomitant intra-articular injuries, and provides direct access to the undersurface of the coracoid process for implant placement. **Methods**: Over the past seven years, we have managed 30 AC separation cases using this arthroscopic approach. **Results**: The distinctive feature of our technique is the use of only two portals—one posterior and one anterosuperior—which proved adequate for optimal visualization and accurate implant positioning. **Conclusions**: In this article, we outline the benefits and limitations of the technique, identify current knowledge gaps, and propose avenues for future clinical research.

## 1. Introduction

Acromioclavicular joint dislocation (ACJD) is among the most common injuries of the shoulder girdle, particularly in young, active individuals. Epidemiological data suggest that ACJ injuries account for approximately 9% of all shoulder girdle traumas, with the majority resulting from contact sports such as rugby, hockey, and American football, as well as high-energy accidents [1]. The Rockwood classification remains the most widely used system for categorizing these injuries, ranging from type I (minor sprain) to type VI (severe displacement with associated soft tissue damage) [2]. While Rockwood types I and II are typically managed conservatively, the optimal treatment for types III–V, and occasionally type VI, remains a subject of debate [3,4,5].

Historically, open surgical techniques have been used to restore anatomical alignment and function. However, these procedures involve larger incisions, carry a risk of hardware irritation, and provide limited visualization of concomitant intra-articular pathology. With the rise of minimally invasive surgery, arthroscopic techniques have emerged as a promising alternative, offering cosmetic advantages, reduced soft tissue trauma, and the ability to simultaneously assess the glenohumeral joint and rotator cuff [6]. Although arthroscopy for AC joint dislocation offers potential advantages, its broader implementation has been limited by technical challenges and specific complications, even though the procedure is considered economically viable [7,8].

Various surgical approaches have been described, including single- and double tunnel techniques coracoclavicular fixation, suspensory button devices, tendon graft augmentations, and combined coracoclavicular and acromioclavicular stabilization. Building on these developments, our study introduces a simplified and practical arthroscopic technique that uses only two portals—one posterior and one anterosuperior—employing suspensory button devices for fixation.

## 2. Diagnosis

**Figure 1:**
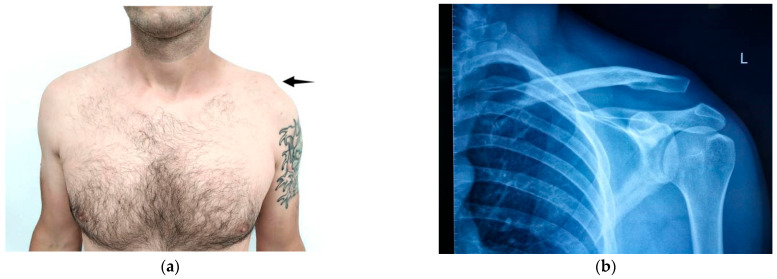
(**a**) Clinical aspect and (**b**) X-ray image of left AC separation.

Diagnosis of AC joint injuries is primarily based on clinical examination and radiographic evaluation. Patients typically report pain localized to the AC joint, which worsens with cross-body movements but can be alleviated with local anesthesia [9]. A visible elevation of the clavicle may be observed, which is temporarily reducible with downward pressure, a phenomenon known as the “piano key sign” (Figure 1).

Radiographs demonstrating a joint space exceeding 5–8 mm confirm the presence of a separation. The Rockwood classification, derived from radiographic assessments, categorizes ACJD into six types based on the degree of displacement [2], serving as a guide for treatment: Types I–II are generally managed conservatively, Types IV–VI require surgical intervention, while Type III remains controversial [5]. The ISAKOS classification further subdivides Type III into IIIA (stable, amenable to nonoperative management) and IIIB (unstable, typically requiring surgery) [10,11,12].

## 3. Treatment Options

More than 150 surgical techniques have been described for AC joint injuries, highlighting the ongoing debate over the optimal approach [13]. Traditional methods for acute injuries, such as K-wires, Bosworth screws, or hook plates, are often associated with complications including arthritis, infection, and the need for hardware removal [14,15].

Suspensory fixation devices have emerged as the preferred choice for acute AC separations, providing anatomical reconstruction, stable fixation, and eliminating the need for hardware removal [16,17]. Initially performed through open surgery, these techniques are now predominantly arthroscopic, offering improved visualization and the ability to simultaneously address glenohumeral lesions.

Chronic injuries, defined as those older than 3–4 weeks [18], require additional stabilization due to limited healing potential. Synthetic ligaments (e.g., carbon fiber, Dacron, LARS, Gore-Tex) aim to restore stability and support biological healing, though the risk of rejection persists [19,20]. Autografts and allografts, by contrast, demonstrate superior biomechanical strength and biological integration [21,22]. Common graft sources include tibialis anterior, flexor carpi radialis, palmaris longus, and peroneus longus tendons, with hamstring tendons (semitendinosus and gracilis) being most frequently used, particularly in chronic cases where biological augmentation is critical.

## 4. Materials and Methods

This retrospective, single-center study, conducted in a non-randomized manner, includes clinical and radiographic follow-up. Its aim is to describe the technique employed by the senior author for managing acromioclavicular (AC) joint separations—a method designed to be reproducible and accessible for surgeons with basic shoulder arthroscopy skills. Our approach to optimal reconstruction focuses on precise reduction of the displaced clavicle, which may occasionally require opening the AC joint to remove a damaged intra-articular disc, followed by arthroscopically assisted reconstruction of the coracoclavicular (CC) ligaments using suspensory fixation devices, with optional augmentation using autografts or synthetic grafts when indicated.

Between January 2018 and January 2025, 30 cases of AC separation were treated using this arthroscopic method. All patients were male, with a mean age of 36.2 years. This distribution reflects epidemiological data from the literature, which report a significantly higher prevalence of acromioclavicular disjunctions in males (male/female ratio was 8.5:1.50) mainly due to increased exposure to contact sports and high-risk traumatic activities [1]. However, we acknowledge that this represents a limitation of the study, as it does not allow for a comparative evaluation of potential differences in presentation and treatment between sexes.

No a priori power analysis was performed, as the sample size was determined by the availability of eligible cases during the study interval. This number, however, provides preliminary data that may serve for future sample size calculations.

In acute cases, suspensory devices alone (TightRope and Dog Bone Button, Arthrex, Naples, FL, USA) were used, while chronic injuries were reinforced with allografts to enhance stability. A key innovation of this technique is the use of only two portals—posterior and anterosuperior—rather than the traditional three, which proved sufficient for visualization and instrument access.

Injuries were classified as acute if managed within three weeks and as chronic if addressed thereafter. Exclusion criteria included patients older than 65 years, those with infections, or lateral clavicle fractures. Patients with Rockwood Type I, II, or low-demand Type III injuries, as well as those who declined surgery, were managed nonoperatively. All surgical procedures were performed by a single senior shoulder surgeon using this customized two-portal arthroscopic approach.

While Rockwood III–V dislocations differ in severity, in our cohort, all patients exhibited pain and functional impairment that warranted surgical treatment. They were managed using the same arthroscopic technique and followed identical postoperative and rehabilitation protocols.

### 4.1. Assesment 

Patients were evaluated at 2 weeks, 6 weeks, 1 year, and 3 years postoperatively. Assessment included AC joint stability, local tenderness, results of the compression and shrug tests, and the range of active and passive shoulder motion. Functional outcomes were analyzed using retrospective pre- and postoperative scores: American Shoulder and Elbow Surgeons (ASES), simple shoulder Test (SST), Specific AC Score (SACS), and Quick Disabilities of the Arm, Shoulder and Hand (Quick DASH). Pain intensity was measured retrospectively with a visual analog scale (VAS) before and after surgery. At the final follow-up, AP radiographs of the AC joint were performed to verify maintenance of CC reduction, and CC distances were compared with early postoperative images by the same doctor. An increase of 5 mm or more was considered a loss of reduction, consistent with reported interobserver variability [7,23]. Any postoperative complications were also documented. Preoperative and postoperative outcome scores were subjected to statistical analysis.

### 4.2. Statistical Analysis

Statistical analysis was performed on pre- and postoperative outcome measures using GraphPad Prism version 10.0.0 (GraphPad Software). The choice of statistical tests was based on data distribution, with both parametric and non-parametric univariate methods employed. Data normality was evaluated using the D’Agostino-Pearson test. For variables with a normal distribution (VAS and SACS), paired t-tests were applied to compare pre- and postoperative values. For SST, ASES, and Quick DASH, which did not meet normality assumptions, comparisons were conducted using the Wilcoxon signed-rank test, a non-parametric alternative.

### 4.3. Surgical Technique

**Figure 2:**
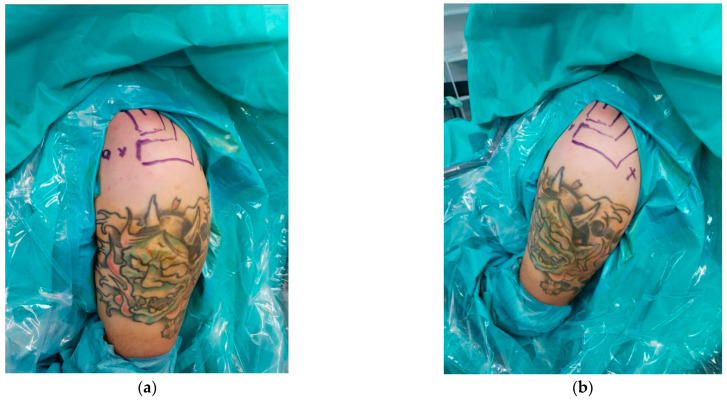
Beach-chair position of the patient during arthroscopic AC repair with bony landmark drawn (**a**) anterior and (**b**) posterior (we used only two portals: standard posterior for visualisation and antero-superior, as a working portal).

The procedure is performed under either general or regional anesthesia, with the patient positioned in the beach-chair position (Figure 2a,b). This position is preferred as it allows rapid conversion to open surgery if complications occur. A fluoroscopy unit is positioned to provide full shoulder visualization, and manual reduction of the AC joint is assessed under fluoroscopic guidance. If reduction is successful, the procedure proceeds arthroscopically. If reduction cannot be achieved, the AC joint is opened to remove any soft tissue obstructing proper clavicle alignment.

After sterile preparation and draping, key bony landmarks are marked with a sterile skin marker. A small incision of 2–3 cm is made approximately 2–3 cm medial to the lateral end of the clavicle. Electrocautery is used to release soft tissues from the anterior to posterior aspects of the clavicle, exposing the bone and facilitating the arthroscopic procedure. In cases where closed reduction is not possible, this incision can be extended toward the AC joint to allow direct access.

The procedure starts with a standard posterior arthroscopic portal to access the shoulder joint. A comprehensive diagnostic assessment is performed to identify any concomitant pathologies, such as labral tears, cartilage lesions, or rotator cuff injuries [23]. Through the posterior portal, the rotator interval is clearly visualized, allowing precise evaluation and planning for subsequent steps of the procedure.

**Figure 3:**
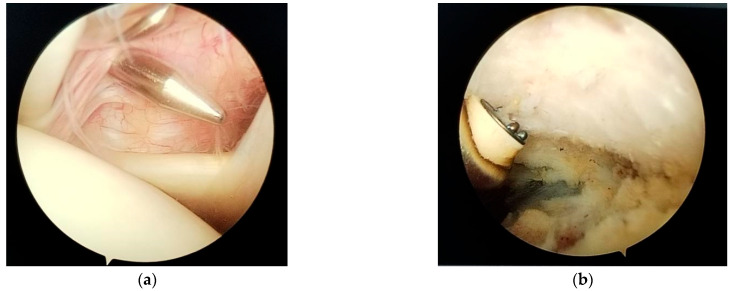
(**a**) anterosuperior portal; (**b**) skeletonization of the inferior part of the coracoid process.

Next, an anterosuperior portal is established using a spinal needle through the rotator interval (Figure 3a). The posterior portal is designated for the arthroscope, and the antero-superior for instruments such as the electrocautery device, and then for the Athrex guide. The anterosuperior portal is typically placed just superior and slightly medial to the biceps tendon within the rotator interval.

For enhanced visualization, especially of the anterior structures, a 70° arthroscope can be used, which may allow the entire procedure to be performed through a single working portal. If a 70° arthroscope is not available, a 30° arthroscope may be used; however, careful debridement of the soft tissue between the anterior glenoid rim and the coracoid process is required.

Next, the inferior surface of the coracoid process is carefully exposed (skeletonized) using electrocautery (Turbovac, Den Bosch, The Netherlands) and a 4.5 mm shaver. It is essential to proceed cautiously during this dissection, avoiding excessive medial extension to prevent injury to the brachial plexus (Figure 3b).

The anterosuperior portal should be positioned as laterally as possible to provide adequate access to the inferior border of the coracoid process. Likewise, the posterior portal is placed slightly lateral to allow a medial view toward the base of the coracoid. The skin incision should be sufficient to accommodate the necessary instruments; however, because working space is often limited, we generally avoid routine use of a cannula.

**Figure 4:**
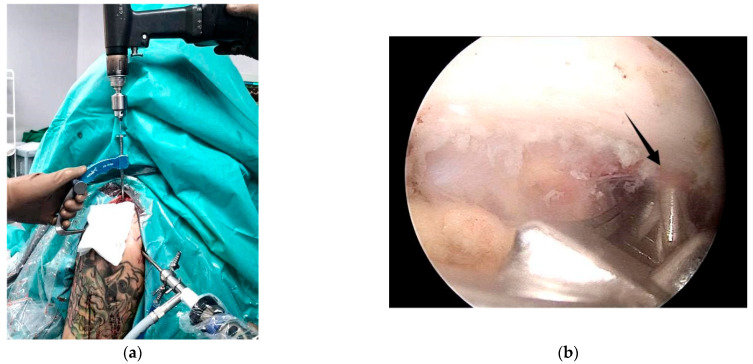
(**a**) the Arthrex guide with the superior limb over the clavicle and (**b**) the inferior limb under the coracoid process.

For the next step, a surgical guide—typically supplied by Arthrex—is employed. Through the anterosuperior portal, the inferior limb of the guide is positioned beneath the coracoid at its junction with the scapular body, commonly referred to as the “knee” of the coracoid, while the superior limb rests over the clavicle. The guide’s cannula is placed centrally on the clavicle, approximately 25 mm from the AC joint (Figure 4).

**Figure 5:**
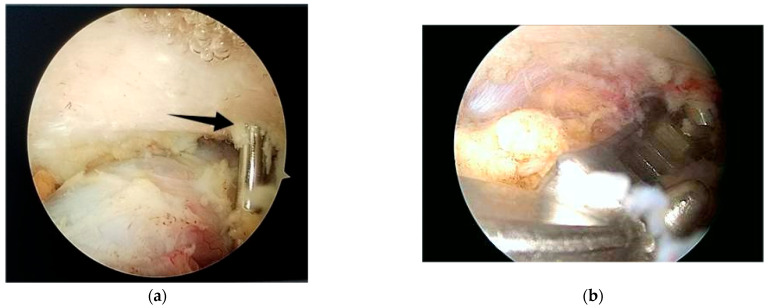
(**a**) The drill exits in the center of the knee of the coracoid; (**b**) the large cannulated guide is inserted over it.

A thin drill is then passed through both the clavicle and the coracoid. Accurate placement is critical, with the drill tip ideally exiting at the center of the coracoid “knee” (Figure 5a), as incorrect positioning significantly increases the risk of coracoid fracture [24].

**Figure 6:**
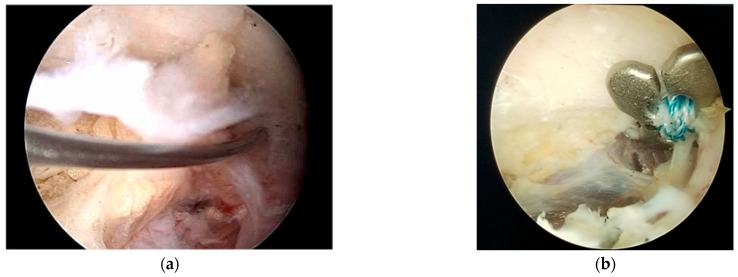
(**a**) A passing suture is advanced through the cannulated guidewire; (**b**) position of the inferior button under the coracoid.

After confirming the drill trajectory, a larger cannulated guidewire is inserted over the initial path (Figure 5b). The initial drill is then removed, and a FiberStick (Arthrex) passing suture is advanced through the cannulated guidewire and retrieved via the anterior portal to facilitate placement of the AC fixation device, which consists of suture tapes and cortical buttons (Figure 6a).

The Dog Bone Button or TightRope device is then passed through the prepared bone tunnels. One cortical button is positioned beneath the coracoid (Figure 6b), while the second button is seated on top of the clavicle.

**Figure 7:**
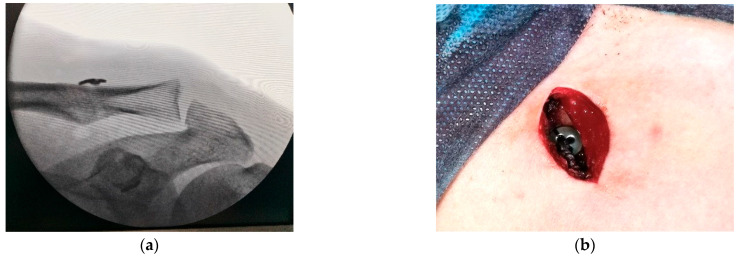
(**a**) superior button over the clavicle; (**b**) final fluoroscopic imaging.

The construct is then tensioned to achieve clavicle reduction and secured with multiple surgical knots (Figure 7a). Proper alignment is finally confirmed using fluoroscopic imaging with a C-arm X-ray system (Figure 7b).

In chronic AC separations, defined as injuries older than 3–4 weeks, augmentation with an autograft, allograft, or synthetic material is required due to the diminished natural healing potential of the joint and ligaments [24,25,26,27]. Our preferred choice is autografts, such as the gracilis tendon harvested from the ipsilateral knee or the palmaris longus tendon from the same side.

With the base of the coracoid process visualized arthroscopically, a cannulated guidewire is inserted from the posterior aspect of the clavicle, exiting on the medial side of the coracoid. A suture is then passed through the guidewire to serve as a shuttle for graft passage. Subsequently, a second cannulated guidewire is inserted from the anterior clavicle, exiting on the lateral side of the coracoid, and the shuttle suture is pulled through.

**Figure 8:**
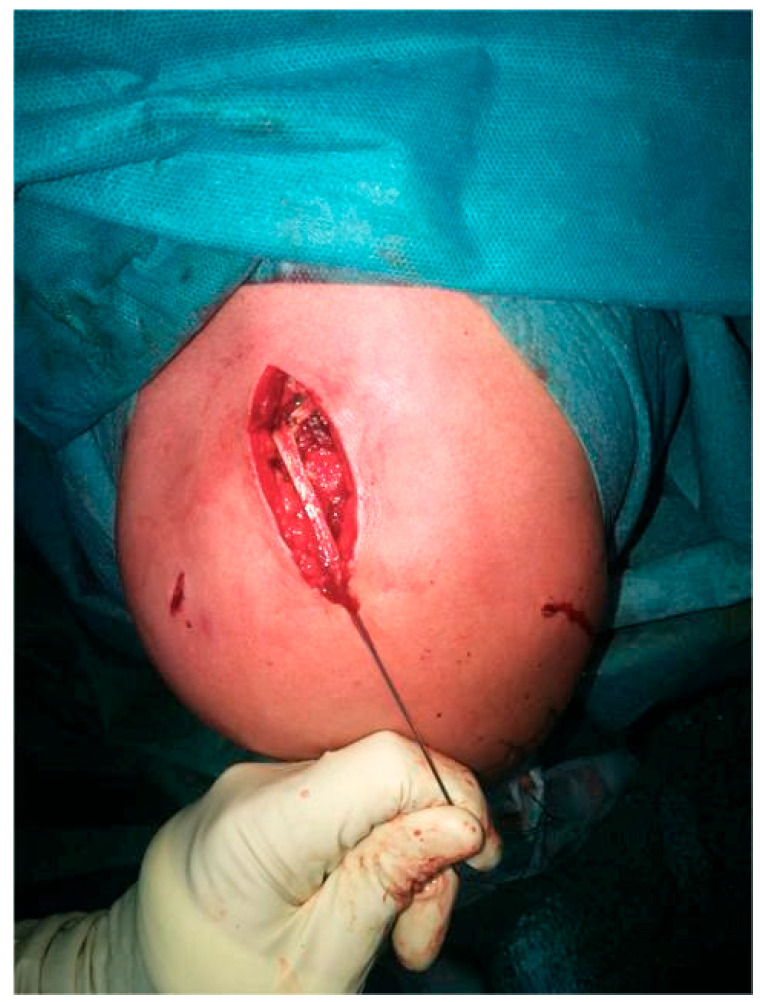
For chronic cases, the gracilis was used to reconstruct the CC and AC ligaments.

Following the previously described AC joint stabilization steps, the graft is attached to the shuttle suture, passed over the clavicle, and looped beneath the coracoid process. The graft ends are tensioned, with the longer limb used to reconstruct the superior portion of the AC joint (Figure 8).

If the AC joint has been surgically opened, small drill holes are created in the distal clavicle and acromion. Sutures are passed through these tunnels and tied securely. The graft can either be threaded through the bone tunnels or simply laid over the AC joint and fixed with sutures.

Fluoroscopic imaging is used at the conclusion of the procedure to confirm proper reduction. The deltotrapezial fascia is closed longitudinally, and the skin is sutured in layers. Postoperatively, the arm is supported in a sling.

### 4.4. Postoperative Care

Postoperatively, patients are allowed to move their arm within a shoulder-level range during the first six weeks to facilitate proper soft tissue healing. After this period, overhead movements may be initiated. Sutures are typically removed 10–14 days after surgery. Follow-up visits are scheduled on the day after surgery, at two weeks, six weeks, six months, one year, and three years.

Rehabilitation begins as early as possible, while respecting the precautions required during the initial healing phase [28]. An immediate postoperative anteroposterior (AP) radiograph is obtained to confirm correct implant placement and reduction, with additional imaging performed a few months later.

**Figure 9:**
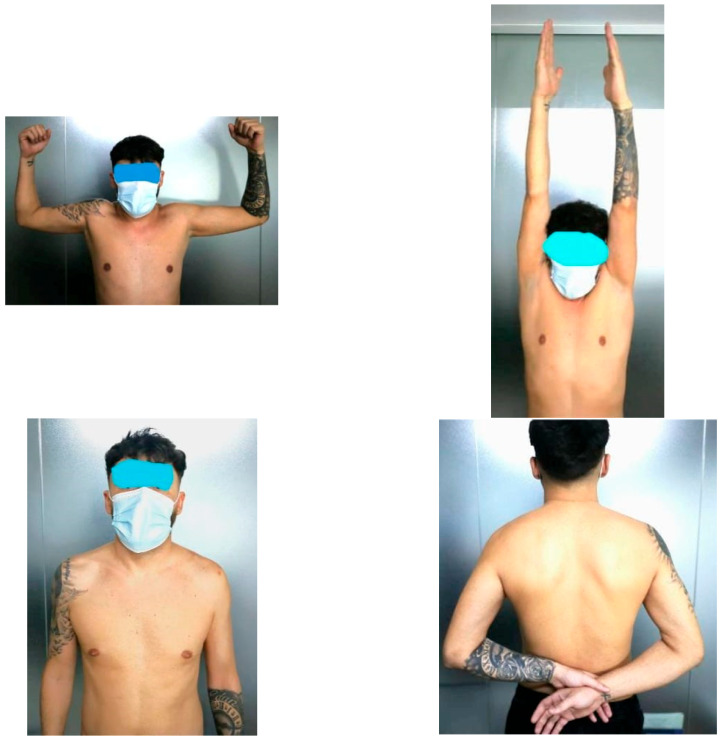
Clinical aspect after 6 weeks from surgery.

Once the full range of motion is achieved, patients may gradually resume previous athletic activities (Figure 9). Muscle strength typically recovers quickly, but lifting heavy objects (over 5 kg) is restricted for several months. High-intensity training is permitted only after six months postoperatively. A schematic overview of the rehabilitation plan is presented in the table (Table 1).

## 5. Results

Thirty patients were accepted in study. All were male with a mean age of 36.2 years (range, 23–65 years; standard deviation [SD], 10.4). According to Rockwood’s classification, there were 14 patients (44.6%) with grade III, 6 (20%) with grade IV, and 10 (33.3%) with grade V injuries. Mean follow-up time was 24.5 months (range, 12–36 months, SD, 4.1). During surgery, we didn’t find significant associated shoulder lesions (Table 2).

In three cases (10%), particularly in lean patients, a small bump was felt at the clavicle incision site due to the presence of the surgical knot. However, this is typically painless and didn’t affect shoulder function. All patients regained full ROM. AC was stable. Three patients had mild AC tenderness (10%), with a positive resisted AC compression test. Two patients had a positive shrug test.

Pre- and postoperative VAS scores was used to evaluate the pain. The mean VAS decreased from 7.6 preoperatively to 1.0 at the final follow-up. Mean preoperative SST and ASES scores were 0.4 and 26.1, respectively, improving at final follow-up to 11.6 for SST and 97 for ASES. The SACS score decreased from 80.3 preoperatively to 5.1 postoperatively. Quick DASH scores improved from 78.9 before surgery to 3.6 at the last follow-up. All pre- versus postoperative differences were statistically significant (*p* < 0.001). The procedure was rated satisfactory by 29 patients (96.6%) (Table 3).

Radiographic evaluation using AP views of the AC joint demonstrated a mean CC distance of 10.6 mm (range 5.8–16.9 mm; SD 2.8 mm) on the first postoperative day. At the final follow-up, the mean CC distance measured 11.2 mm (range 7.5–15.1 mm; SD 1.9 mm). Considering that interobserver variability in prior studies is around 5 mm, this suggests there was no clinically meaningful loss of reduction between short- and long-term postoperative assessments. In our series, three patients (10%) showed a CC distance increase greater than 5 mm (6.3, 7.1, and 8.2 mm), though none exceeded 10 mm. All instances corresponded to a radiographic relapse to grade II, without any clinical impact. Four patients had radiological findings of osteoarthritis, but without clinical implications (Table 4).

No other complications were observed: there were no infections, no revisions, and no implant changes were required. Except for one case that experienced mild pain at the clavicular button (which improved after 18 months), all other patients had a favorable outcome. The follow-up assessment of functional outcomes and pain is summarized in the table (Table 5).

## 6. Discussion

This study aimed to emphasize the advantages and long-term reliability of an arthroscopic technique for managing acute grade III, IV, and selected grade V acromioclavicular (AC) dislocations, with particular attention to long-term outcomes. Although limited by a small patient cohort and the absence of a non-arthroscopic control group using the same implant, the findings indicate that our minimally invasive approach provides significant clinical benefits. The procedure capitalizes on the biological healing potential of the native coracoclavicular (CC) ligaments, making it most suitable for acute injuries within three weeks; older injuries typically require open procedures with allograft augmentation [24,25,26,27,28].

The arthroscopic technique demonstrated low complication rates: no infections were observed, and loss of reduction occurred in only three patients (10%), outperforming the 18.9% average reported in previous studies. The revision rate was 0%, substantially better than alternative approaches. The use of only two working portals—one posterior and one anterosuperior—minimized soft tissue trauma, facilitating faster recovery, reduced pain, and lower infection risk. Precise arthroscopic visualization of the coracoid “knee” enabled accurate guidewire placement, reducing the risk of perioperative fractures. Both clavicular and coracoid tunnels were limited to a 4 mm diameter to enhance safety.

Arthroscopy offers multiple advantages over open surgery. Small incisions and advanced instruments reduce tissue damage, while the magnified surgical field allows detailed assessment and simultaneous treatment of associated pathologies, such as ligament tears, labral injuries, or rotator cuff lesions. Accurate placement of the fixation device is critical; arthroscopy permits precise guidewire positioning at the strongest part of the coracoid, improving reduction stability and minimizing fracture risk—a benefit not always achievable with open techniques.

Despite these benefits, arthroscopic AC reconstruction requires advanced surgical expertise. Surgeons must carefully avoid neurovascular injury, particularly to the brachial plexus and musculocutaneous nerve, and perform conservative soft tissue debridement to protect the conjoint tendon while maintaining visualization. Correct guidewire placement is essential to prevent clavicle fractures. Knotless systems, such as those developed by Arthrex, further enhance patient comfort and cosmetic outcomes [29].

In acute dislocations, reduction of the clavicle and stabilization with suspensory devices, such as the TightRope or Dog Bone Button, is generally sufficient. In chronic cases, additional augmentation with autografts, allografts, or synthetic materials may be necessary. When damaged AC joint cartilage impedes reduction, preoperative fluoroscopic guidance can assist planning. If reduction is feasible, the implant can be placed arthroscopically; otherwise, surgical debridement and AC ligament reconstruction are required to prevent instability.

Overall, the arthroscopic technique described in this study—particularly the use of only two portals—proved to be highly effective, safe, and tissue-sparing, demonstrating clear advantages over conventional open procedures.

Clinical outcomes in our cohort showed marked improvements across all validated parameters. Pain, as assessed by the VAS score, decreased substantially from 7.6 ± 1.8 preoperatively to 1.0 ± 1.5 postoperatively (*p* < 0.001). Shoulder function improved significantly, with SST increasing from 0.4 ± 0.6 to 11.6 ± 0.6 and ASES from 26.1 ± 18 to 97 ± 6 (both *p* < 0.001). Similarly, SACS score decreased from 80.3 ± 15 to 5.1 ± 7, indicating restoration of joint stability, while QuickDASH improved from 78.9 ± 20 to 3.6 ± 6, reflecting recovery of upper limb function (both *p* < 0.001). These results confirm that arthroscopic reconstruction not only reduces pain but also enables substantial functional recovery, return to daily activities, and return to sports in the majority of patients. The magnitude of these improvements is consistent with previous reports in the literature, supporting the reliability of arthroscopic techniques in the management of Rockwood type III–V acromioclavicular dislocations [26,27,28].

This study has several limitations. The cohort was relatively small (*n* = 30) and all participants were male, which may limit generalizability. No control group was included for comparison with open surgery or alternative techniques. All procedures were performed at a single center by one surgeon, and follow-up duration varied slightly among patients. Despite these limitations, the study provides useful insights into the outcomes and rehabilitation following arthroscopic acromioclavicular joint reconstruction.

## 7. Conclusions

Acromioclavicular joint separations commonly occur in young, active individuals and require careful management, particularly in severe cases (Rockwood IV–VI) or selected type III injuries, such as in athletes. Optimal outcomes are achieved through anatomic restoration of the joint, often using suspensory fixation devices like the TightRope or Dog Bone Button, sometimes supplemented with autografts, allografts, or synthetic ligaments.

Our technique utilizes a minimally invasive arthroscopic approach with only two working portals, minimizing soft tissue trauma while ensuring precise implant placement. Arthroscopy provides additional benefits, including enhanced visualization of the coracoid process, reduced risk of fracture or infection, smaller incisions, the ability to address associated shoulder injuries, and improved cosmetic results.

When performed by experienced surgeons and combined with a structured rehabilitation program, this method achieves excellent functional recovery, with most patients regaining mobility within weeks. The initial six-week period is critical for protection, and return to high-intensity sports is generally recommended after six months. Patient adherence to postoperative instructions is essential for successful outcomes.

## Figures and Tables

**Table 1:** Postoperative and Rehabilitation Care.

Weeks	Tasks
1–6	An arm sling is applied Ice packs are applied to the shoulder Mobilization of the elbow and wrist started Mobilization of the arm up to shoulder level Optimal analgesia is provided to the patient
6–12	Rehabilitation progresses from passive to active range-of-motion exercises. Isometric exercises are initiated first, followed by progressive strengthening of the rotator cuff. Mobilization of the elbow and wrist is maintained throughout.
12+	Patients may begin proprioceptive and strengthening exercises.
6 Months	The patient is allowed to return to sports

**Table 2:** Patient Demographics and Injury Characteristics.

Parameter	Value
Lot size	30
Age	36.2 ± 10.4 (years)
Follow-up time	24.5 ± 4.1 (months)
Sex	Male
ACDJ grade III	14 (44.6%)
ACDJ grade IV	6 (20%)
ACDJ grade V	10 (33.3%)

Abbreviations: ACDJ = Acromioclavicular Disjunction.

**Table 3:** Functional Outcome Scores (Preoperative vs. Postoperative).

Parameter	Preoperative	Postoperative	*p*-Value
VAS	7.6 ± 1.8	1.0 ± 1.5	<0.001
SST	0.4 ± 0.6	11.6 ± 0.6	<0.001
ASES	26.1 ± 18	97 ± 6	<0.001
SACS	80.3 ± 15	5.1 ± 7	<0.001
QuickDASH	78.9 ± 20	3.6 ± 6	<0.001

Abbreviations: VAS = Visual Analog Scale; SST = Simple Shoulder Test; ASES = American Shoulder and Elbow Surgeons Score; SACS = Subjective Assessment of the Clinical Shoulder; QuickDASH = Quick Disabilities of the Arm, Shoulder, and Hand.

**Table 4:** Postoperative Complications.

Parameter	Cases (%)
CC loss of reduction >5 mm	3 cases (10%)
Age	0 cases
CC loss of reduction >10 mm	24.5 ± 4.1 (months)
AC osteoarthritis	4 cases (13.3%)

Abbreviations: CC = Coracoclavicular; AC = Acromioclavicular.

**Table 5:** Pain Assessment.

Follow-Up (Months)	Patients Available	VAS (Mean ± SD)	SST (Mean ± SD)	ASES (Mean ± SD)	SACS (Mean ± SD)	QuickDASH (Mean ± SD)
Preoperative	30	7.6 ± 1.8	0.4 ± 0.6	26.1 ± 18	80.3 ± 15	78.9 ± 20
1	30	5.8 ± 1.6	3.5 ± 1.5	40 ± 12	55 ± 14	60 ± 18
3	29	3.9 ± 1.5	6.8 ± 1.4	60 ± 12	30 ± 11	32 ± 14
6	28	2.2 ± 1.4	9.5 ± 1.0	78 ± 10	15 ± 9	14 ± 9
12	27	1.5 ± 1.3	11. ± 0.7	90 ± 8	7 ± 7	3.6 ± 6
24	27	1.0 ± 1.5	11.6 ± 0.6	97 ± 6	5.1 ± 7	3.6 ± 6

Abbreviations: VAS: Visual Analog Scale for pain; SST: Simple Shoulder Test; ASES: American Shoulder and Elbow Surgeons score; SACS: Shoulder and Acromioclavicular Score; Quick DASH: Disabilities of the Arm, Shoulder, and Hand (short version).

## Data Availability

The data presented in this study are available in references [1,2,3,4,5,6,7,8,9,10,11,12,13,14,15,16,17,18,19,20,21,22,23,24,25,26,27,28,29].
